# Physical Demands of Women's Soccer Matches: A Perspective Across the Developmental Spectrum

**DOI:** 10.3389/fspor.2021.634696

**Published:** 2021-04-16

**Authors:** Jason D. Vescovi, Elton Fernandes, Alexander Klas

**Affiliations:** ^1^Faculty of Kinesiology and Physical Education, University of Toronto, Toronto, ON, Canada; ^2^Graduate School of Exercise Science, University of Toronto, Toronto, ON, Canada

**Keywords:** women's soccer, match demands, running distance, metabolic power, acceleration

## Abstract

Female soccer players are exposed to specific physical demands during matches, which vary according to the standard of play. Existing studies have largely focused on quantifying the distances covered for professional and international level players. This approach is limited in scope regarding the broader aspects around physical demands and is detached from development pathway models. An understanding of the demands across all standards will provide valuable insights about appropriate player development and help ensure physical readiness for the demands of the sport. The aim of this perspective paper is to describe the physical demands experienced during women's soccer matches across the developmental spectrum. A combination of evidence from the literature and data from the author's research (JDV) is presented. Specifically highlighted are the trends for locomotor distances, acceleration and deceleration frequency, and metabolic power metrics for youth (≤U17), college (NCAA/U20), professional (domestic) and international standards of women's soccer. In addition, the changes in match demands between levels of play are used to help illustrate gaps that must be overcome in order to successfully achieve physical readiness to compete at higher levels. The evidence demonstrates the importance of training appropriate attributes to prepare female soccer players who are striving to play at progressively higher standards.

## Introduction

Female soccer players are exposed to specific physical demands during matches, which vary according to the level/standard of play. To date, researchers and sport scientists have generally focused on the highest-level teams (i.e., elite) in order to develop strategies aimed at optimizing the physical readiness of these players and ultimately winning League titles, World Championships, or Olympic medals. Solely focusing match analysis at this level certainly has benefits for broadening our knowledge of the demands experienced by the best players (and teams), but is simultaneously disconnected from player development models, where gaps in technical, tactical, and fitness capabilities are routinely evaluated. Therefore, a comprehensive understanding of the physical demands of women's soccer matches across a wider range of standards is an essential element for enhancing player development pathways. There has been increased attention at some lower levels (i.e., U21, college), but there is still a paucity of research available describing the physical demands of youth matches.

Despite the recent interest and popularity in describing the physical demands of women's soccer matches, there are two substantial challenges to overcome for organizations, coaches, and sport science practitioners when examining published studies. First, outcomes between (i.e., video, GPS) and within (e.g., GPS 1, 5, and 10 Hz) systems are not interchangeable (Buchheit and Simpson, 2017), so some latitude is warranted when attempting to compare studies. Second, there is currently no consensus regarding the thresholds used for establishing discrete bands for key performance indicators (e.g., locomotor distances) (Bradley and Vescovi, 2015; Park et al., 2019). As a result, it is difficult to make direct comparisons within the published literature and subsequently create a cohesive view across the entire developmental spectrum for match demands. Recognizing these limitations, the aim of the current perspective paper is to describe match demands of women's soccer across the developmental spectrum in two ways. First, by presenting and comparing (when possible) published studies and second, by including data from the author (JDV) that uniquely spans youth, college, professional, and international women's soccer matches. The benefit of including these data is that they were collected using identical technology (5 Hz GPS) and applied the same thresholds for all reported variables–thus, direct comparisons can be made across all standards of play within this dataset.

## Methods

A literature search of Pubmed and Google Scholar was conducted using a combination of the following terms: women's soccer, match demands, running distance, metabolic power, acceleration, deceleration, youth soccer, college soccer, professional soccer, elite soccer, and international soccer. Additionally, the references of identified studies were searched for other citations not found in the electronic search. Both studies and reports were included that described the locomotor distances, acceleration and deceleration profiles, and/or metabolic power metrics for women's soccer matches at any standard of play. Despite the limitations of comparing various data collection technologies, papers were selected regardless of the methodology used to quantify match demands (i.e., video, GPS, etc.). To simplify and help facilitate comparisons of locomotor metrics between published studies, we focused on total distance, movement rate (distance per minute), and high-intensity distances (inclusive of sprinting) since the latter has been identified as a key indicator that differentiates between levels of play in women's soccer (Mohr et al., 2008; Andersson et al., 2010). We recognize that positional differences in physical demands exist; however, an examination of this component was beyond the scope of the current perspective.

Data from the author's (JDV) previous research is included, which contains locomotor distance, acceleration and deceleration frequencies, and metabolic power metrics. These particular data are presented for descriptive purposes only and do not represent an experimental study; therefore, statistical analyses were not applied. Players competed across four standards: youth (U15 to U17), college, professional (PRO), and elite international (INT). All matches were played on regulation sized soccer fields with referees. Youth players (U15 = 21, player-matches = 21; U16 = 69, player-matches = 85; U17 = 32, player-matches = 32) were involved in a high-level tournaments or talent identification camps with varying match durations (35–45 min halves) depending on the specific age-group. Matches at the other three standards had 45 min halves. College players (*n* = 51, player-matches = 71) competed in regular season NCAA Division I matches. Professional players (*n* = 83, player-matches = 205) competed in regular season (domestic) matches for a professional league. In addition to domestic players, each team included several international players from their respective national teams. Elite international players (*n* = 12, player-matches = 39) were from a top ranked FIFA women's national team and competed in “friendly” matches against other ranked FIFA women's national teams.

Players wore a GPS unit (SPI Pro 5-Hz, GPSports, Canberra, Australia) that is valid and reliable for measuring sprint distance and speed (Petersen et al., 2009; Waldron et al., 2011). Between 8 and 12 satellites were available for signal transmission (Jennings et al., 2010). Horizontal-dilution-of-precision values > 4 were automatically removed by the Team AMS software, which is below the maximum value (50) reported to result in inaccurate outcomes (Witte and Wilson, 2004). A digital watch that received satellite time identified the start and end of each half, as signaled by the referee's whistle. Data were extracted using the manufacturer software (GPSports, Team AMS R1 2015.10J) for analysis. The outcomes are presented in the current paper using the following thresholds for locomotor distance (Bradley and Vescovi, 2015), metabolic power (Osgnach et al., 2010), and acceleration/deceleration (manufacturer default setting):

- Locomotor distance: ≤ 6.0 kph (Zone 1), 6.1–8.0 kph (Zone 2), 8.1–12.0 kph (Zone 3), 12.1–16.0 kph (Zone 4), 16.1–20.0 kph (Zone 5), and > 20.0 kph (Zone 6)
- Metabolic power: ≤ 10.0 W/kg (Zone 1), 10.1–20.0 W/kg (Zone 2), 20.1–35.0 W/kg (Zone 3), 35.1–55.0 W/kg (Zone 4), and >55 W/kg (Zone 5)
- Acceleration and deceleration: 1.80–3.60 m/s^2^ (Zone 1), 3.61–5.40 m/s^2^ (Zone 2), and 5.41–7.20 m/s^2^ (Zone 3)

## Locomotor Demands

Locomotor demands are the most popular metrics in soccer and have been widely reported for several decades. Some of the pioneering work in this space included manually coding video of recorded games (Bangsbo et al., 1991), whereas today there are automated video systems permanently installed in stadiums as well as GPS technology that afford teams a mobile option that can be used almost anywhere. These technological advances have enabled the continued expansion of data collection, which ultimately allow practitioners to capture data more easily and report the distances covered within various velocity bands.

### Youth

To date, there are five studies describe the demands of female youth soccer matches (Barbero-Alvarez et al., 2008; Vescovi, 2014; Orntoft et al., 2016; Ramos et al., 2019; Harkness-Armstrong et al., 2020). The youngest age groups to be reported is U11-U12; however, the modified structure (see Table 1) makes direct comparisons to the literature impossible (Barbero-Alvarez et al., 2008; Orntoft et al., 2016). Still, the 20 and 50-min games resulted in ~1,600 and 3,963 m of total distance, respectively, and a corresponding movement rate of about 80 m/min (Barbero-Alvarez et al., 2008; Orntoft et al., 2016), which is expectedly lower than movement rates for older age groups. A study during a youth national championship tournament (domestic) reported the demands of U15 to U17 players competing in typical match configurations (11v11 for 80–90 min) (Vescovi, 2014). The U15 players covered 6,961, 458, and 76 m for total, high-intensity (15.6–20.0 kph) and sprinting (>20 kph) distances, respectively, which were lower than the distances reported for U16 (8,024, 611, 185 m) and U17 (8,558, 658, and 235 m) age-group matches. These age-group differences persisted even after accounting for match duration with lower movement rates for U15 (86 m/min) compared to U16 and U17 (93–95 m/min) players. Talent pathway league matches for U14 and U16 teams showed average total distances of 7,148 and 7,679 m, respectively, with 217 and 247 m of high-intensity running (>19.0 kph) (Harkness-Armstrong et al., 2020). During the women's U17 South American championships (international), the players for Brazil covered 8,270, 485, and 191 m for total, high-intensity (15.6–20 kph), and sprint distances (>20 kph), respectively (Ramos et al., 2019). In general, there seems to be comparable total distances and movement rates for the same age groups, but the two studies using the same velocity thresholds showed high-intensity distances were about 24% lower during the international event (676 m) (Ramos et al., 2019) than the domestic event (893 m) (Vescovi, 2014). This difference could be attributable to the GPS technology used (5 vs. 10 Hz), environmental conditions, as well as game tactics (e.g., formation, style of play, etc.). Overall, it seems progressively greater movement rates occur across age-group youth soccer matches and is likely a reflection of improved physical capacities of players (Mujika et al., 2009) while holding match contextual factors constant.

**Table 1:** Velocity thresholds and distances for high-intensity running and sprinting across standards.

			**High-intensity**		**Sprint**	
**References**	**Competition standard**	**Method**	**Velocity (kph)**	**Distance (m)**	**Velocity (kph)**	**Distance (m)**
**Youth**						
Barbero-Alvarez et al. (2008)	U12 (7v7, 50 min game)	GPS (1 Hz)	13.1–18.0	228	>18.0	21
Harkness-Armstrong et al. (2020)	U14 (35 min half)	GPS (10 Hz)	> 19.0	1,530	>22.5	29
	U16 (40 min half)			1,695		53
Orntoft et al. (2016)	U11 (7v7 & 8v8, 20 min game)	GPS (5 Hz)	16.0–20.0	34–63	> 20.0	0
Ramos et al. (2019)^*^	U17 International	GPS (10 Hz)	15.6–20.0	485	> 20.0	178
Vescovi (2014)	U15 (40 min half)	GPS (5 Hz)	15.6–20.0	458	> 20.0	76
	U16 (40 min half)			611		185
	U17 (45 min half)			658		235
**College**						
Alexander (2014)^*^	Division I	GPS (10 Hz)	15.1–18.0	527	> 18.0	362
Gentles et al. (2018)	Division II	GPS (5 Hz)	15.1–25.0	~1,140	> 25.0	~80
Jagim et al. (2020)	Division III	GPS (10 Hz)	15.0–19.0	739	> 19.0	282
McCormack et al. (2015)	Division I	GPS (10 Hz)	13.0–22.0	~2,283	> 22.0	NR
McCormack et al. (2014)	Division I	GPS (10 Hz)	13.0–22.0	1,586	> 22.0	NR
McFadden et al. (2020)	Division I	GPS (10 Hz)	15.0–19.0	~800	> 19.0	~400
Ramos et al. (2019)^*^^‡^	U20 International	GPS (10 Hz)	15.6–20.0	660	> 20.0	202
Sausaman et al. (2019)	Division I	GPS (10 Hz)	15.0–18.0	1,014	> 18.0	428
Strauss et al. (2019)	University	GPS (10 Hz)	> 15.5	~336		
Turczyn (2018)	University	GPS (15 Hz)	16.0–19.9	~680	> 20.0	~250
Vescovi and Favero (2014)^*^	Division I	GPS (5 Hz)	15.5–20.0	776	> 20.0	250
Wells et al. (2015)	Division I-Reg season	GPS (10 Hz)	16.0–22.0	557	> 22.0	86
	Division I-Post season			603		85
**Professional and elite**						
Andersson et al. (2010)	Professional	Video-S	15.0–18.0	1,330	18.0–25.0	221
	Elite International			1,530		256
Bradley et al. (2014)	Professional	Video-M (25 Hz)	12.0–18.0	2,374	> 18.0	777
Bradley and Scott (2020)	Elite International (2015)	Video-M (20 Hz)	13.0–23.0	~2,493	> 23.0	~140
	Elite International (2019)	Video-M (20 Hz)		~2,563		~181
Datson et al. (2017)	Professional	Video-M	19.8–25.1	608	> 25.1	168
DeWitt et al. (2018)	Professional	GPS (10 Hz)	> 17.8	570	> 22.7	NR
Hewitt et al. (2014)	Elite International	GPS (5 Hz)	12.0–19.0	2,407	> 19.0	338
Julian et al. (2020)	Professional (1st/2nd Div)	GPS (5 Hz)	16.7–19.9	~567	>19.9	~342
Krustrup et al. (2005)	Professional	Video-S	15.0–18.0	1,310	18.0–25.0	160
Mara et al. (2017b)	Professional	Video-M (25 Hz)	12.2–19.0	2,452	> 19.0	615
Martínez-Lagunas and Scott (2016)	Elite International (2011)	Video-M (25 Hz)	16.0–20.0	846	>20.0	485
	Elite International (2015)	Video-M (20 Hz)		868		472
Martínez-Lagunas et al. (2016)	Professional (2nd Div)	GPS (5 Hz)	16.0–20.0	671	>20.0	290
	Professional (4th Div)			515		162
Meylan et al. (2017)	Elite International	GPS (10 Hz)	16.5–20.0	~542	> 20.0	~250
Mohr et al. (2008)	Professional	Video-S	15.0–18.0	1,300	18.0–25.0	380
	Elite International			1,680		460
Moraleda et al. (2021)	Professional	GPS (5 Hz)	>15.0	1,108		
Nakamura et al. (2017)	Professional	GPS (5 Hz)		NR	> 20.0	284
Principe et al. (2021)	Professional	GPS (10 Hz)	16.0–20.0	~599	>20.0	~303
Ramos et al. (2017)	Elite International	GPS (10 Hz)	15.6–20.0	744	> 20.0	304
Ritschard and Tschopp (2012)	Elite International (2011)	Video-M (25 Hz)	18.1–21.0	395	>21.0	290
Scott et al. (2020a)^*^	Professional (domestic)	GPS (10 Hz)	12.5–22.5	2,746	> 22.5	119
	Professional (internat)			2,834		150
Scott et al. (2020b)	Professional	GPS (10 Hz)	12.5–22.5	2,799	> 22.5	122
Trewin et al. (2018a)	Elite International	GPS (10 Hz)	> 16.5	~873	> 20.0	NR
Trewin et al. (2018b)	Elite International	GPS (10 Hz)	> 16.5	~855	> 20.0	NR
Vescovi and Falenchuk (2019)	Professional	GPS (5 Hz)	16.1–20.0	~756	> 20.0	~351

* *weighted average across positions using mean values*;

‡ *same sample used in 2019 paper; NR, not reported*.

*Video-S (single camera); Video-M (multi-camera)*.

### College

Research examining the demands of women's college soccer matches has gained attention during the past few years. An important distinction to note about NCAA matches is that they do not follow international standards for substitutions. So, researchers tend to either include players that competed in full matches (which limits the sample size) or “create” 90-min matches from multiple players (which tends to alter movement rates). This is evident from a study comparing Division I regular season and post-season matches, which showed a 10% increase in total distance with a corresponding 6.5% decrease in movement rate (Wells et al., 2015). Nevertheless, total distances reported for NCAA Division I (~9,000–9,900 m) (McCormack et al., 2014; Vescovi and Favero, 2014; Sausaman et al., 2019), Division II (~10,000) (Gentles et al., 2018), Division III (~9,600–9,800 m) (Jagim et al., 2020) as well as Canadian University matches (~8,800–9,600 m) (Turczyn, 2018) are fairly similar, with subsequent movement rates of ~100–110 m/min. Despite slightly different velocity thresholds used to define high-intensity running, it seems that when the velocity band spans only 3–4 kph the amount of distance covered is within a range of ~600–800 m (Vescovi and Favero, 2014; Wells et al., 2015; Ramos et al., 2017; Turczyn, 2018; Jagim et al., 2020) with a notable exception reaching ~1,000 m (Sausaman et al., 2019). For sprint distances, increasing the lower limit velocity threshold from 18 to 19 kph (~280–420 m) (Alexander, 2014; Sausaman et al., 2019; Jagim et al., 2020; McFadden et al., 2020) to 20 kph (~200–250 m) (Vescovi and Favero, 2014; Ramos et al., 2017) and 22 kph (<100 m) (Wells et al., 2015) has the expected reduction of reported distances.

Investigators have also examined the impact of contextual factors (e.g., altitude, match frequency, etc.) on locomotor demands in college matches. Moderate altitude (1,839 m) had a negative effect on total (121 vs. 106 m/min) and high-intensity (28 vs. 25 m/min) movement rates (Bohner et al., 2015), suggestive that hypoxic conditions adversely impacted locomotor activity. When college soccer matches end in a draw, the teams play two 10-min extra-time periods. The additional 20 min result in a 22–23% increase in total distances for extra-time matches compared with 90-min matches (Williams et al., 2019). Surprisingly, the total distances covered during extra-time were equivalent (~1,100 m) between players who competed in the entire match or only a portion of the match. The NCAA soccer match schedule can be considered congested, where multiple games are oftentimes played with minimal days off in between. One study demonstrated that regular Friday and Sunday matches throughout the season resulted in lower total (120 vs. 106 m/min) and high-intensity (25 vs. 22 m/min) movement rates during the second game (McCormack et al., 2015). Similarly, Canadian women's soccer matches showed a ~13% reduction in high-intensity (16–20 kph) and sprint (>20 kph) distance when games were on back-to-back days (Turczyn, 2018). Interestingly, there was no impact of poor sleep on match demands in these players (Turczyn, 2018). In contrast to the impact of match schedule, no differences were observed for high-speed or sprint distances between NCAA Division I regular-season and playoff matches (Wells et al., 2015), suggestive that players were able to continue playing with similar intensity throughout an entire season. Overall, these studies highlight how contextual factors might impact game demands for female college soccer players and the need to monitor these metrics in order to manage the physical demands players experience during the season.

### Professional and International

There is substantially more evidence describing the locomotor demands of elite female soccer with a fairly even distribution between professional (domestic) (Krustrup et al., 2005; Mohr et al., 2008; Andersson et al., 2010; Bradley et al., 2014; Martínez-Lagunas et al., 2016; Datson et al., 2017; Mara et al., 2017b; Nakamura et al., 2017; DeWitt et al., 2018; Vescovi and Falenchuk, 2019; Julian et al., 2020; Scott et al., 2020a; Moraleda et al., 2021; Principe et al., 2021) and international matches (Mohr et al., 2008; Andersson et al., 2010; Ritschard and Tschopp, 2012; Hewitt et al., 2014; Martínez-Lagunas and Scott, 2016; Trewin et al., 2018a,b; Ramos et al., 2019; Bradley and Scott, 2020; Scott et al., 2020a,b).

The average total distances reported among professional (~8,200–11,000 m) and international (~9,300–11,000 m) level matches are generally similar. The majority of studies have demonstrated movement rates between 100 and 120 m/min (Krustrup et al., 2005; Mohr et al., 2008; Andersson et al., 2010; Bradley et al., 2014; Hewitt et al., 2014; Datson et al., 2017; Mara et al., 2017b; Trewin et al., 2018a,b; Julian et al., 2020; Scott et al., 2020b) with only a few showing movement rates below 100 m/min (Martínez-Lagunas et al., 2016; DeWitt et al., 2018; Moraleda et al., 2021; Principe et al., 2021) and one above 120 m/min (Datson et al., 2017). Interestingly, only the top finishing teams in the 2015 and 2019 FIFA Women's World Cups had movement rates that were aligned (105–113 m/min) with the general consensus from the literature, whereas the bottom finishing teams were between 86 and 94 m/min (Bradley and Scott, 2020). Additionally, there was a sizeable gap in movement rate between German teams in the 2nd division (~104 m/min) and 4th division (~91 m/min) (Martínez-Lagunas et al., 2016). The differences between top and bottom teams within a given standard of play (i.e., divisions in a professional league or international events like the World Cup) could be the result of contextual factors (e.g., lower-level teams making tactical decision to largely play defense). It is also possible these outcomes highlight supportive evidence for the link between fitness levels and distances covered during women's soccer matches (Krustrup et al., 2005).

There is a substantial range describing the high-speed running and sprinting distances in elite women's matches, which is directly attributable to the wide variety of thresholds used to define these particular metrics (see Table 1). For example, some studies have used 12.0–12.5 kph as the lower limit for a given threshold (e.g., high-intensity running) (Bradley et al., 2014; Hewitt et al., 2014; Mara et al., 2017b; Scott et al., 2020a,b), but the upper limit has ranged between 18.0 and 22.5 kph. Subsequently, the reported distances vary from ~2,300–2,450 m (Bradley et al., 2014; Hewitt et al., 2014; Mara et al., 2017b) when using 18.0 kph, compared with ~2,800 m being captured as a result of using 22.5 kph (Scott et al., 2020a,b). Three studies have examined both professional and international women's matches; despite similar total distances between standards (within their respective studies) (Mohr et al., 2008; Andersson et al., 2010; Scott et al., 2020a), two studies reported 15–29% more high-intensity running during international matches (Mohr et al., 2008; Andersson et al., 2010), whereas the other found only a 3% difference (Scott et al., 2020a). The wider velocity zone (12.5–22.5 kph) (Scott et al., 2020a) may have impacted the outcomes because smaller differences in distance are seen between standards at slower speeds (e.g., 6–16 kph), thus potentially washing out an effect that was observed when using a smaller velocity zone (15–18 kph) (Mohr et al., 2008; Andersson et al., 2010). On the other hand, it is possible that differences between international and professional matches dissipate when players at both levels compete together. Thus, elevated international match demands may be the result of contextual factors inherent to the competition itself (i.e., higher stakes, greater motivation), rather than as unique physiological characteristics of international level players. Lastly, the influence of natural (~660 m) and synthetic (~770 m) turf on high-intensity running during women's matches has also been reported (Vescovi and Falenchuk, 2019) and demonstrates how this contextual factor might impact match demands at this standard of play.

Sprinting distances between studies also varies widely because of the different velocity thresholds. Several studies have used >20 kph and found sprint distances between ~250 and ~350 m per match (Martínez-Lagunas et al., 2016; Meylan et al., 2017; Nakamura et al., 2017; Trewin et al., 2018a,b; Ramos et al., 2019; Vescovi and Falenchuk, 2019; Julian et al., 2020; Principe et al., 2021), with substantially smaller distances shown (120–180 m) when higher thresholds are used (22.5–25.1 kph) (Datson et al., 2017; Bradley and Scott, 2020; Scott et al., 2020a,b). These differences between studies are expected since the impact of implementing various high-velocity thresholds on these locomotor distances has been previously demonstrated in professional women's matches (Vescovi, 2012; Bradley et al., 2014). Nevertheless, players competing in international matches have 16–26% more sprint distances than during professional matches (Mohr et al., 2008; Andersson et al., 2010; Scott et al., 2020a). An examination of the previous three FIFA Women's World Cups indicates nearly identical sprint distances between 2011 and 2015 (485 vs. 472 m; using >20 kph) (Martínez-Lagunas and Scott, 2016), but a 21% increase from 2015 to 2019 (~558 vs. ~677 m; using >19 kph) (Bradley and Scott, 2020). Taken together and despite the difficulty of making direct comparisons between published studies, greater high-intensity demands are evident at the highest standard.

### Developmental Perspective

**Figure 1:**
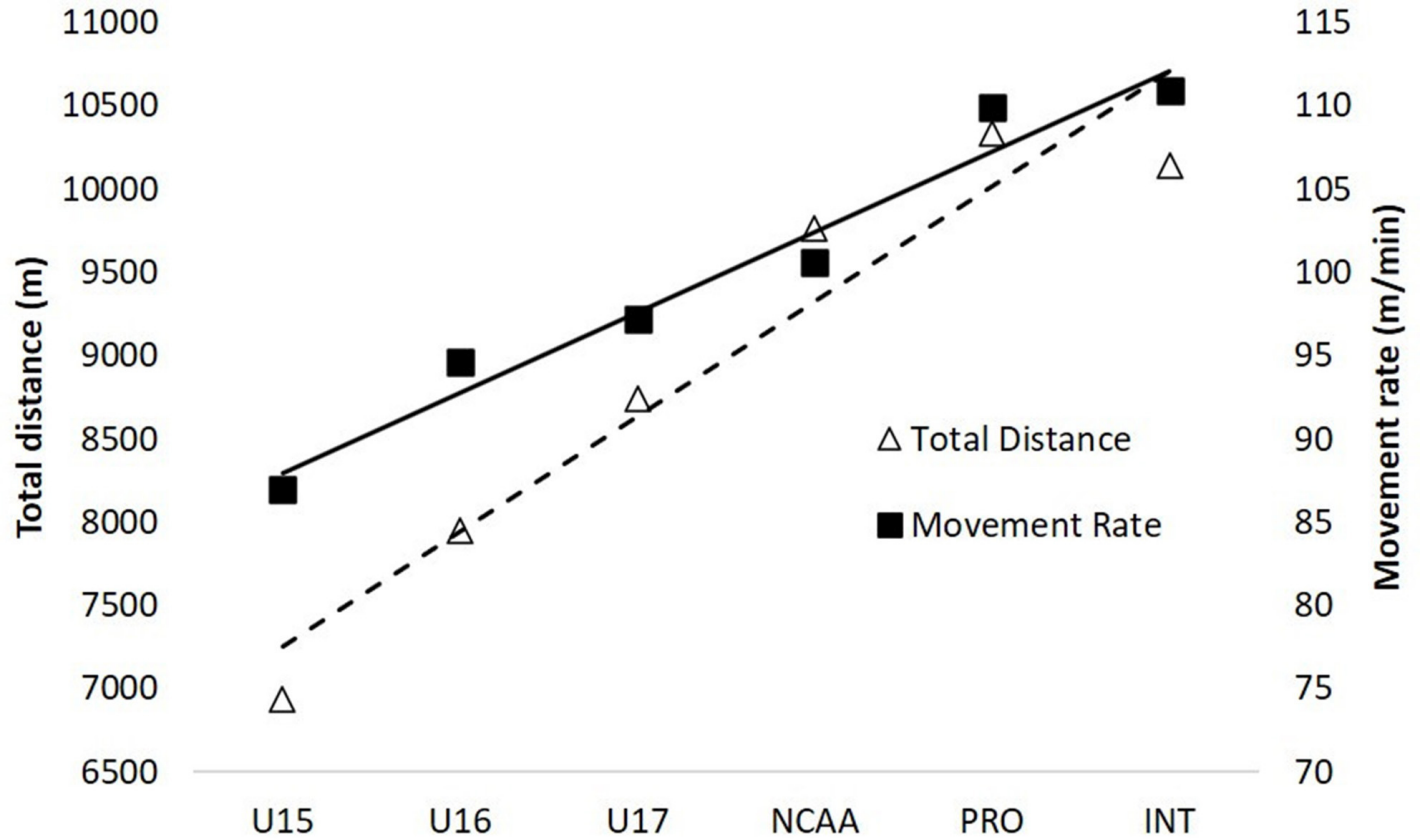
Average total distance and movement rate across standards. Data from author (JDV).

**Figure 2:**
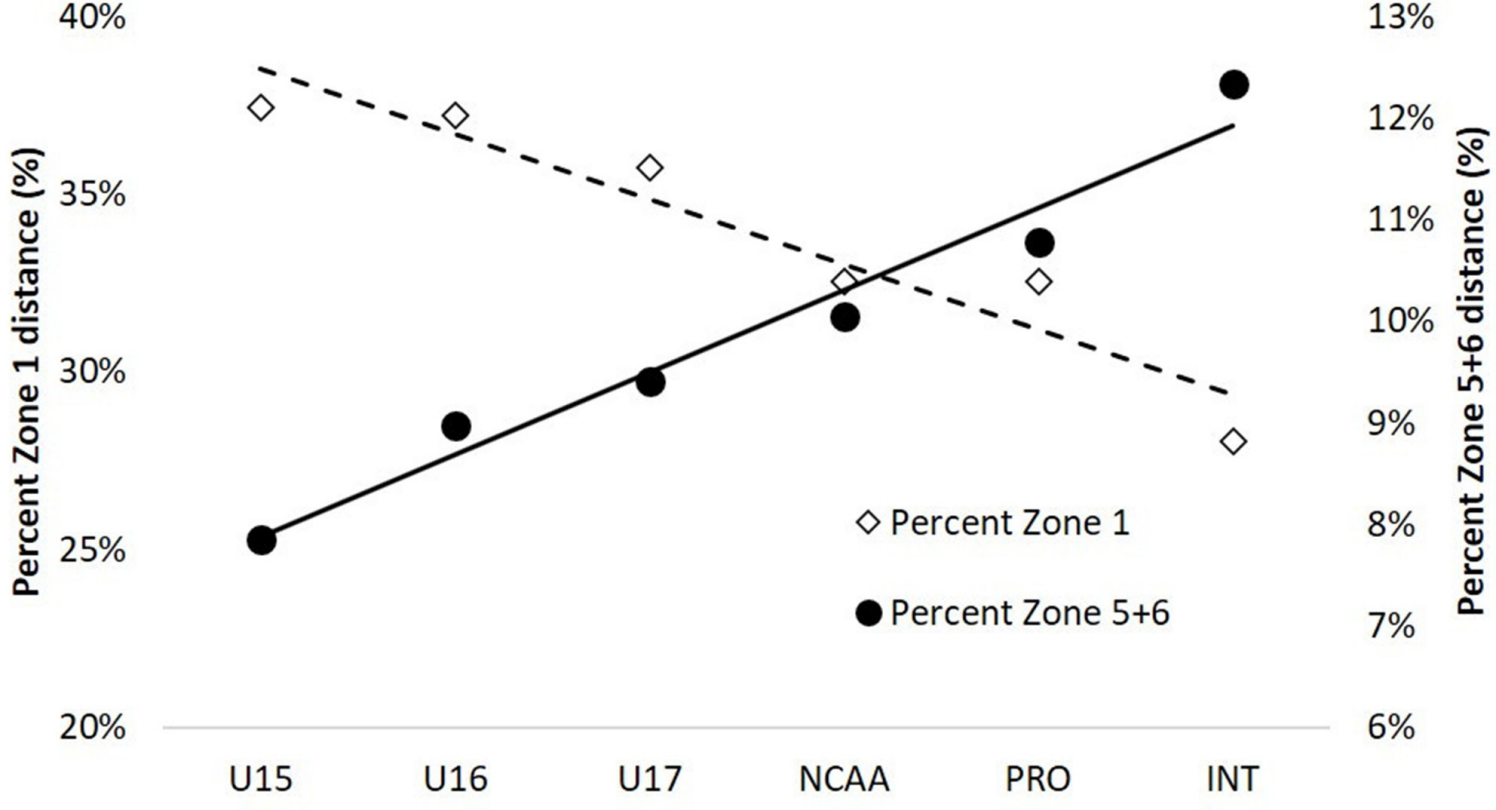
Average percent distance of Zone 1 and Zone 5+6 across standards. Data from author (JDV).

Table 2 displays the total distance, movement rate and distances in each velocity band across the developmental spectrum. In general, total match distances are aligned with data from the literature for the respective cohorts and shows a strong linear increase from youth (~7.0–8.7 km) to professional and international matches (~10 km) (Figure 1). This relationship remained even after taking match duration into account, although after the NCAA a plateau of movement rate occurred for professional (domestic) and international matches. The movement rates for professional and international matches are similar to the top teams that competed in the 2011 (106–120 m/min) (Ritschard and Tschopp, 2012), 2015 (108–113 m/min) (Martínez-Lagunas and Scott, 2016), and 2019 (105-110 m/min) FIFA Women's World Cup (Bradley and Scott, 2020) tournaments. As a proportion of total distance, Zone 2 (~12–13%) and Zone 3 (~27.5–29.5%) remained fairly constant between all standards of play. However, there was a larger change in the relative distances for lower (Zone 1) and higher (Zone 5+6) speed movements, demonstrating that younger players perform greater proportions of walking and smaller proportions of high-intensity movement compared to higher standards (Figure 2). So, young players will be exposed to greater demands progressing through age-groups and then again if they progress to play at the college level. The greater demands through youth and into college will be somewhat connected to increased match duration, but more important, also linked with higher match tempos. College athletes seeking to compete at even higher standards will be required to have ~10% faster movement rates in order to match the tempo of professional and international players. This will result from a substantially greater percent change for high-intensity distances (Zones 5+6) compared with total distance. For example, the relative changes between U17 and NCAA Division I is 12% for total distance and 19% for high-intensity running. Similarly, 6% and 13% increases are evident when transitioning between Division I and professional matches for total and high-intensity distance, respectively. These are critical pieces to understand when designing the physical preparation component of player development models.

**Table 2:** Locomotor distances and movement rate across standards.

	**Match**	**<6 kph**	**6–8 kph**	**8–12 kph**	**12–16 kph**	**16–20 kph**	**>20 kph**	**>16 kph**	**Total**	**Rate**
	**Duration (min)**	**Zone 1 (m)**	**Zone 2 (m)**	**Zone 3 (m)**	**Zone 4 (m)**	**Zone 5 (m)**	**Zone 6 (m)**	**Zone 5+6 (m)**	**Distance (m)**	**(m/min)**
U15	80 (2)	2,597 (368)	838 (193)	1,996 (395)	958 (226)	465 (111)	79 (61)	545 (141)	6,936 (335)	87 (4)
U16	84 (1)	2,957 (358)	896 (179)	2,168 (469)	1,211 (365)	562 (179)	150 (115)	713 (206)	7,946 (869)	94 (11)
U17	90 (0)	3,124 (328)	1,031 (162)	2,461 (610)	1,306 (456)	609 (163)	213 (174)	823 (281)	8,746 (928)	97 (10)
NCAA	97 (4)	3,178 (279)	1,251 (185)	2,898 (487)	1,455 (307)	744 (205)	237 (121)	981 (309)	9,762 (774)	101 (8)
PRO	94 (2)	3,363 (369)	1,276 (258)	2,851 (484)	1,728 (471)	752 (184)	361 (191)	1,113 (288)	10,332 (877)	109 (9)
INT	91 (2)	2,846 (247)	1,242 (114)	2,977 (308)	1,827 (318)	837 (172)	414 (170)	1,251 (276)	10,144 (546)	111 (6)

*Values are mean (SD). Data from author (JDV)*.

## Acceleration and Deceleration Demands

Quantifying the distance covered and movement rate of soccer matches only describes a portion of the demands experienced by players since the intermittent nature of the game requires frequent changes of speed. The positive (acceleration) and negative (deceleration) changes in speed impose additional demands on the body than when moving at a constant velocity (Osgnach et al., 2010). These changes in speed may be brief and not meet the duration (e.g., >1 s) or speed (e.g., >20 kph) requirements that would result in the activities being labeled as high-velocity running activities within GPS systems. Nevertheless, they are still high-intensity actions based on acceleration (Akenhead et al., 2013; Mara et al., 2016; Nakamura et al., 2017). Therefore, it is important for practitioners to give consideration to the quantity and intensity of accelerations and decelerations when examining match demands of women's soccer.

### Youth

There is a single study describing the acceleration profile for youth women's soccer matches (Ramos et al., 2019). The physical demands of seven matches from the U17 National Brazilian team demonstrated that, on average, players performed 150–200 accelerations (>1 m/s^2^) and 85–122 decelerations (> −1 m/s^2^) during an international event.

### College

Several research groups have described acceleration profiles (Ramos et al., 2017, 2019; Jagim et al., 2020) as well as accelerometer derivative metrics (e.g., player load) (Wells et al., 2015; Gentles et al., 2018; Strauss et al., 2019) for college age-group matches. The accelerometer derivatives might be useful metrics because they can account for movements such as jumping; however, these do not directly reflect changes in horizonal speed and thus are not considered here.

Categorizing movements into specific bins demonstrated the vast majority of accelerations and decelerations were low intensity (± 0.5–1.99 m/s^2^, 953 vs. 1,010, respectively), when compared with moderate (± 2.00–2.99 m/s^2^, 64 vs. 69, respectively), and high-intensity (± 3.00–50.0 m/s^2^, 10 vs. 17, respectively), counts for Division III NCAA matches (Jagim et al., 2020). During a U20 international tournament players performed 172–196 accelerations and 108–145 decelerations (>1 m/s^2^) (Ramos et al., 2019); however, these values were substantially reduced when the threshold was increased to >2 m/s^2^ (13–17 and 11–25, respectively), in the same group (Ramos et al., 2017).

### Professional and International

Several studies have reported acceleration (Meylan et al., 2017; Trewin et al., 2018a,b; Principe et al., 2021) and deceleration profiles (Mara et al., 2017a; Ramos et al., 2019; Principe et al., 2021) for elite female soccer players. Three studies were conducted by the same group and implemented the same definition (>2.26 m/s^2^) to quantify the frequency of accelerations that occurred during matches (Meylan et al., 2017; Trewin et al., 2018a,b). In general, the number of accelerations performed was about 1.8/min (~162 for 90 min match) (Meylan et al., 2017; Trewin et al., 2018b). They also demonstrated contextual factors (hot environment had lowest count = 1.73/min or ~156; draws vs. lower teams had highest count = 2.07/min or ~186) influenced the number of accelerations performed (Trewin et al., 2018a,b).

A few studies described both acceleration and deceleration profiles (Ramos et al., 2019; Moraleda et al., 2021; Principe et al., 2021). The Brazilian women's national team was monitored during the Rio 2016 Olympic Games and had average acceleration and deceleration counts ranging from 201–218 to 161–182 per match, respectively (Ramos et al., 2019). These outcomes demonstrate acceleration counts are about 16–38% higher when using a substantially lower threshold (1 m/s^2^) (Ramos et al., 2019) than reported above. Professional Brazilian players had substantially lower acceleration and deceleration frequencies (also used >1 m/s^2^ - ~155 and ~157, respectively) (Principe et al., 2021) than their national team counterparts, highlighting the distinction between these two standards of play. Another study implemented a threshold of 2 m/s^2^ but uniquely described accelerations and decelerations by the starting and finishing speed associated with the movement and classified the frequency counts in six different zones (Mara et al., 2017a). They reported a total of 423 and 430 accelerations and decelerations, respectively, with the majority of them (~250 each) having a low starting and ending speed (<3.4 m/min). Interestingly, this study found substantially greater counts of accelerations and decelerations in elite women's matches (about double) despite using a threshold that was between other research groups (1.0 vs. 2.0 vs. 2.26 m/s^2^). Perhaps the video system (25 Hz) is more sensitive than GPS technology (10 Hz) for quantifying these match demands. When monitoring acceleration counts for elite matches practitioners need to consider match-to-match variation (12–21%) (Meylan et al., 2017; Trewin et al., 2018a).

### Developmental Perspective

The acceleration and deceleration profiles shown in Table 3 highlight several key features across the developmental spectrum. First, more than 95% of accelerations and 85% of decelerations occurred between 1.8 and 3.6 m/s^2^. These thresholds are the manufacturer defaults, nevertheless the skewed distribution suggests that alternative values are likely needed if practitioners want to qualify these metrics as low, moderate and high-intensity. Improved quantification of frequency counts might require assessment of acceleration ability across the developmental spectrum to identify appropriate thresholds. Second, the total accelerations for professional and international matches (145–158 or 1.6–1.8/min) appear somewhat aligned with previous studies using GPS technology, despite implementing different thresholds (1 and 2.26 m/s^2^) (Meylan et al., 2017; Trewin et al., 2018a,b; Ramos et al., 2019). It is unclear what could cause this but is likely a result of subtle differences in vendor software calculations. Lastly, the largest change in acceleration (~34%) and deceleration (~26%) frequency occurs between youth and NCAA matches. This highlights a substantial component for player development pathways aimed at athletes making the transition from high school to college.

**Table 3:** Acceleration and deceleration frequencies across standards.

	**Match**	**1.8–3.6 m/s^**2**^**	**3.6–5.4 m/s^**2**^**	**5.4–7.2 m/s^**2**^**	**Accel**	**1.8–3.6 m/s^**2**^**	**3.6–5.4 m/s^**2**^**	**5.4–7.2 m/s^**2**^**	**Decel**
	**Duration (min)**	**Accel 1 (n)**	**Accel 2 (n)**	**Accel 3 (n)**	**Total (n)**	**Decel 1 (n)**	**Decel 2 (n)**	**Decel 3 (n)**	**Total (n)**
U15	80 (2)	98 (19)	2 (2)	0	101 (20)	103 (17)	9 (6)	1 (1)	113 (18)
U16	84 (1)	109 (33)	3 (2)	0	112 (34)	112 (21)	12 (5)	1 (2)	125 (26)
U17	90 (0)	109 (31)	2 (2)	0	112 (31)	114 (24)	13 (6)	1 (1)	129 (29)
NCAA	97 (4)	144 (29)	5 (3)	0	149 (31)	141 (32)	20 (8)	2 (2)	163 (38)
PRO	94 (2)	145 (26)	5 (3)	0	151 (27)	144 (28)	21 (8)	2 (2)	167 (32)
INT	91 (2)	158 (23)	6 (4)	0	164 (25)	146 (22)	23 (8)	3 (2)	172 (27)

*Values are mean (SD). Data from author (JDV)*.

## Metabolic Power Demands

The introduction of metabolic power (di Prampero et al., 2005) and subsequent application to soccer match analysis (Osgnach et al., 2010) has integrated acceleration and deceleration data to define additional metrics. This method has been suggested to better represent match demands than relying upon velocity based demands alone, especially for high-intensity work (Gaudino et al., 2013). The outcomes include distances within various metabolic power bands (similar to establishing velocity bands for locomotor distances) as well as the energetic cost (internal load metric). An important note, unlike velocity thresholds, the originally proposed metabolic power thresholds (Osgnach et al., 2010) have been consistently applied in the literature, which are: <10 (low), 10–20 (moderate), 20–35 (high), 35–55 (elevated), and > 55 (maximal) W/kg. Although updated algorithms have been developed to improve the accuracy of this method (di Prampero and Osgnach, 2018; Osgnach and di Prampero, 2018), it should be noted that studies reporting on metabolic power in women's soccer are scarce and have applied the original approach (di Prampero et al., 2005).

### Youth

As of this writing, there are no published studies examining the metabolic power demands of youth female soccer matches.

### College

Two studies have examined metabolic power in women's college soccer matches (Wells et al., 2015; Williams et al., 2019) but described limited outcome variables despite the ability to quantify several more (Osgnach et al., 2010). In one study, the mean high-metabolic power distance (>20 W/kg) in NCAA Division I matches was 1,839 and 440 m for regulation and extra-time, respectively (Williams et al., 2019). The total for that study (2,279 m total) is similar to unpublished data from NCAA matches (2,126 m–Table 3) that also includes stoppage-time (mean match duration, 97 min). An important consideration for practitioners is that stoppage and extra-time can substantially elevate the amount of high-metabolic power distance (Williams et al., 2019), therefore recovery strategies from those matches become more important, especially if there is little time before the next match as often is the case with the NCAA soccer schedule.

Using this method (Osgnach et al., 2010) also allows for an estimation of energetic demands. One study reported a 10% increase in energy expenditure between regular season (34 kj/kg) and post-season (38 kj/kg) college matches which was linked to an additional 700 m of total distance (Wells et al., 2015). The energetic demands were greater (48 kj/kg) in another group of Division I players (Williams et al., 2019) and even higher for NCAA teams in Table 4 (53 kj/kg). Although all were NCAA Division I teams, the ones included in Table 4 had a high national ranking (e.g., six of nine teams ranked top 30 and three ranked top 10). Similar to the differences described for locomotor movement rates between top and bottom teams in FIFA Women's World Cup (Bradley and Scott, 2020) there are likely variations in the tempo of play across NCAA Division I that could subsequently impact energetic demands of these matches. Furthermore, the implementation of the metabolic power methodology could be modified by commercially available GPS systems (Williams et al., 2019) in order to have a proprietary competitive advantage, which could have also influenced the reported outcomes between studies (Terziotti et al., 2018). Additionally, when converting the relative energetic demands from these studies into calorie expenditure (520–770 kcal) (Wells et al., 2015; Williams et al., 2019), they are substantially lower than values obtained using heart rate derived equivalents (~1,100–1,400 kcal) (Jagim et al., 2020; McFadden et al., 2020), thus it does not appear the outcomes from various methods can be used interchangeably.

**Table 4:** Metabolic power distances and load across standards.

	**Match**	**<10 W/kg**	**10–20 W/kg**	**20–35 W/kg**	**35–55 W/kg**	**>55 W/kg**	**>20 W/kg**	**Equivalent**	**Metabolic**
	**Duration (min)**	**Zone 1 (m)**	**Zone 2 (m)**	**Zone 3 (m)**	**Zone 4 (m)**	**Zone 5 (m)**	**Zone 3+ (m)**	**Distance (m)**	**Load (kj/kg)**
U15	80 (2)	3,926 (312)	1,555 (274)	881 (133)	342 (60)	147 (47)	1,370 (178)	8,411 (452)	39.1 (2)
U16	84 (1)	4,402 (277)	1,853 (492)	1,026 (260)	395 (107)	179 (97)	1,600 (400)	9,349 (1,136)	43.4 (5)
U17	90 (0)	4,789 (231)	2,021 (528)	1,197 (289)	461 (99)	178 (86)	1,835 (416)	10,170 (1,195)	47.2 (6)
NCAA	97 (4)	5,124 (276)	2,415 (466)	1,378 (257)	522 (100)	225 (66)	2,126 (372)	11,569 (997)	53.7 (5)
PRO	94 (2)	5,144 (293)	2,435 (436)	1,460 (279)	573 (103)	274 (80)	2,307 (392)	11,617 (1,507)	53.9 (7)
INT	91 (2)	4,935 (170)	2,681 (303)	1,595 (209)	624 (81)	306 (73)	2,527 (299)	11,745 (1,121)	51.9 (11)

*Values are mean (SD). Data from author (JDV)*.

### Professional and International

To date there are two published studies that include metabolic power demands for female players. Both studies evaluated professional domestic match play and included the same cohort of players from the WPS league (Vescovi, 2016; Vescovi and Falenchuk, 2019). Playoff matches showed greater mean metabolic power (10.2 W/kg) than regular season matches (9.2 W/kg), which corresponded to ~23, 26, and 29% more relative distance covered in high (19.4 vs. 15.8 m/min), elevated (7.2 vs. 5.7 m/min) and maximal (2.2 vs. 1.7 m/min) metabolic power categories, respectively (Vescovi, 2016). There was little impact on metabolic power metrics when examining various contextual factors (i.e., home vs. away, natural vs. artificial turf, and match outcome) (Vescovi and Falenchuk, 2019). The only notable difference was greater high-metabolic power distance when matches were played on artificial turf (16.3 m/min) than on natural turf (14.4 m/min). When the top three categories are taken together (>20 W/kg) the distances covered on natural and artificial turf were ~2,070 and 2,313 m (Vescovi and Falenchuk, 2019), which are greater than the value previously described for college matches (regulation-time 1,839 m) (Wells et al., 2015).

The energetic demands of players competing at higher standards have also been described. During a modified match structure (3 × 20 min), female players had a relative energetic load of 37 kj/kg (~2,400 kj) (Mara et al., 2015a). Even higher values have been reported from professional regular-season and post-season matches (51–58 kj/kg) (Vescovi, 2016; Moss et al., 2020). This equates to ~900 kcal expenditure during professional women's soccer matches, which is greater than measured values (~744 kcal) reported for professional German players during a 90-min training game (Martínez-Lagunas, 2013). However, the overall movement demands were lower (total distance ~ 7,230 m and distance >16 kph ~631 m) than values typically observed during regulation matches and so lower energy expenditure values would be expected. Nonetheless, these data highlight the overall energetic needs for female players is likely between 750 and 900 kcal per match.

### Developmental Perspective

The data provided in Table 4 fills some of the gaps identified in the literature surrounding metabolic power and also includes a derived metric, equivalent distance. The equivalent distance is a way to express the distance an athlete would have traveled at a steady pace on grass by using the total energy expended during the entire match (Osgnach et al., 2010). The ratio of equivalent distance to total distance (called equivalent distance index–EDI) has been previously defined for convenience to be ~1.20 (Osgnach et al., 2010). The equivalent distance and its index may be metrics of interest because they represent the overall volume and metabolic intensity a player experiences during a match, respectively (di Prampero and Osgnach, 2018; Osgnach and di Prampero, 2018). It is evident that there are steadily increasing values for several metrics such as metabolic load, movement rate, and equivalent distance from youth and into the NCAA matches, which then seem to plateau at higher standards. Similar to Table 2, the percent change among standards for high-metabolic power distance (Zone 3+ >20 W/kg) is substantially larger than the corresponding percent change between levels for equivalent distance (9–17 vs. 1–14%, respectively). The reason this occurred is unknown, but since these metrics take into account acceleration/deceleration, their distribution might offer insights into potential links. Currently, the skewed distribution of this dataset obstructs an understanding on this topic - perhaps applying different acceleration/deceleration thresholds would be better suited to investigate this in the future. Nonetheless, metabolic power outcomes provide supportive evidence for giving attention to developing the ability to perform greater amounts of high-intensity effort across the developmental spectrum.

## Practical Considerations and Applications

The data presented highlights the physical demands of women's soccer matches across the developmental spectrum. This information can be used by clubs, leagues and federations for player development within and between levels of play. It could also be used for return to play protocols for injured players during the rehabilitation process. It is beyond the scope of this paper to detail the specific ways to go about incorporating this into the daily training environment and rehabilitation settings. However, coaches and fitness practitioners can likely focus on two overarching objectives with respect to effectively using information on the physical demands of women's matches. The first way is to help players achieve the match demands within their current standard. For example, players on a particular team or teams within a given level (e.g., U15, NCAA Division III) will demonstrate a range of physical match demands for any of the metrics described above. Improving the physical fitness qualities of players/teams that are at the lower end of the range can subsequently have a positive impact on performance during matches. The second focus for coaches and practitioners is to prepare athletes/teams who are looking to transition to the next higher standard (i.e., a player going from college to professional, or a team being promoted from a lower to higher professional division). In these circumstances, the physical preparation must be targeted at the demands for the higher level with care taken to implement a periodized plan over a sufficient amount of time to elicit the desired (beneficial) physiological adaptations.

A general heuristic often followed in endurance training is for athletes to perform ~2.0–2.5 times the competition distance as total weekly training volume. Translated to women's soccer, that would mean if total distances during matches were ~7 km (youth), ~9 km (college), or ~11 km (professional/elite), then total weekly volume should roughly be 14–18, 18–22, and 22–28 km, respectively. These theoretical targets for total weekly training distance seem to be somewhat aligned with what has been reported for professional teams (~16–22 km, exclusive of matches) (Mara et al., 2015b; Moraleda et al., 2021). Please note, the ratio (2.0–2.5X) would only be applied to total volume since evidence-based recommendations on other metrics (i.e., sprint distance, volume of accelerations, metabolic power) do not currently exist. These distances could be programmed into the weekly training sessions and incorporated directly into practice with soccer-specific drills and small-sided games that target particular attributes of interest (e.g., very short maximal accelerations [<5 m], achievement of maximal velocity [15–20 m], etc.). This type of approach enables technical, tactical and physical components to be developed simultaneously and reduces the need for additional (off-field) work.

## Moving Forward

There has been a steady increase in the number of published studies describing the physical demands of women's soccer matches. The advancements in video capture systems and GPS technology have enabled the expansion of insights about a broad spectrum of movement demands. Still, there are gaps specific to women's soccer that have been noted by others (Martínez-Lagunas et al., 2014) and need to be addressed. First, there is a lack of standardized thresholds for quantifying locomotor distances as well as acceleration and deceleration profiles. This prevents a unified understanding of match demands across the developmental spectrum. The use of physiological (Bradley and Vescovi, 2015; Trewin et al., 2018a) and mathematical (Park et al., 2019) approaches have been suggested but still have not been embraced (e.g., different thresholds implemented in previous three Women's World Cup events) (Ritschard and Tschopp, 2012; Martínez-Lagunas and Scott, 2016; Bradley and Scott, 2020). Second, there is a tremendous gap in research describing the physical demands of youth soccer matches (≤U17). In order to provide comprehensive training recommendations for developmental pathways additional attention is required. Work has been initiated in this area (Harkness-Armstrong et al., 2020), but needs to continue through National Sport Organizations and professional academies that have the necessary resources to monitor players within their ecosystem, but effort is also required by researchers to partner with women's youth domestic leagues in order to broaden the scope of understanding. Lastly, there is limited data about the metabolic power metrics in women's soccer, which now exist in most commercially available GPS systems. Therefore, if metabolic power provides insights beyond velocity-based movement demands, then researchers should begin to include these outcomes in published studies. Overall, the direction of research in women's soccer is very promising and continued advancements to fill these gaps will ensure that better, evidence-based recommendations are applied to the physical developmental component of female player pathway models.

## Data Availability Statement

The datasets presented in this article are not readily available because of pre-existing legal agreements. Requests to access the datasets should be directed to Dr. Jason Vescovi.

## Ethics Statement

The studies involving human participants were reviewed and approved by York University, Office of Research Ethics. Written informed consent to participate in this study was provided by the participants' legal guardian/next of kin.

## Author Contributions

JDV was responsible for manuscript concept, data collection, writing, and revision of the paper. EF and AK were responsible for conducting the literature search, writing, and revision of the paper. All authors contributed to the article and approved the submitted version.

## Conflict of Interest

The authors declare that the research was conducted in the absence of any commercial or financial relationships that could be construed as a potential conflict of interest.
